# Postpartum depression among mothers attending postnatal clinics in Bole sub-city, Addis Ababa, Ethiopia, in the post-COVID-19 era: A cross-sectional study

**DOI:** 10.1371/journal.pgph.0006553

**Published:** 2026-06-05

**Authors:** Dagmawit M. Tasew, Biruk Tesfahun. Mengistie, Chernet T. Mengistie, Mikiyas G. Teferi, Iyosaphit E. Essay, Nuhamin W. Zeleke, Samrawit W. Emamnew, Saron D. Tadesse, Abirham A. Minayehu, Natnael Tesfu Legesse, Ashenafi Z. Berhe

**Affiliations:** 1 Myungsung Medical College, Myungsung Specialized Comprehensive Hospital, Addis Ababa, Ethiopia; 2 School of Medicine, College of Health Sciences, Addis Ababa University, Addis Ababa, Ethiopia; 3 Department of Public Health, Myungsung Specialized Comprehensive Hospital, Addis Ababa, Ethiopia; PLOS: Public Library of Science, UNITED STATES OF AMERICA

## Abstract

Postpartum depression (PPD) harms maternal and child health. Evidence showed elevated PPD during the COVID-19 pandemic, but post-pandemic data from low-resource urban settings are limited. We measured PPD prevalence and its correlates among mothers attending postnatal clinics in Bole sub-city, Addis Ababa, Ethiopia, during the post-COVID-19 period (data collection: 1 March–30 April 2024). An institution-based cross-sectional study enrolled 410 mothers who delivered 2 weeks–1 year prior, attending five primary health centres. Participants were selected using systematic random sampling, with proportional allocation based on client flow across five primary health centers. Depressive symptoms were screened with the Edinburgh Postnatal Depression Scale (EPDS); a cutoff ≥11 defined probable PPD. Data were entered in EpiData and analysed in SPSS. Bivariate analyses (p < 0.25) identified candidates for multivariable logistic regression; adjusted odds ratios (AOR) with 95% confidence intervals (CI) and p < 0.05 indicated statistical significance. A total of 410 mothers participated (mean age 28.8 ± 4.7 years). The prevalence of postpartum depression (PPD) was 20.0% (82/410; 95% CI: 16.4–24.1%). In multivariable logistic regression analysis, primiparity (AOR = 7.83; 95% CI: 1.29–47.71), unplanned pregnancy (AOR = 2.49; 95% CI: 1.25–4.95), poor partner support (AOR = 13.23; 95% CI: 3.08–56.94), low social support (AOR = 3.31; 95% CI: 1.13–9.74), and induction of labor (AOR = 2.73; 95% CI: 1.13–6.60) were independently associated with higher odds of postpartum depression. One in five mothers screened positive for PPD in this post-COVID-19 urban primary-care sample. First-time mothers and those with limited partner or social support, unplanned pregnancies, or induction of labour are at elevated risk. Integrating routine PPD screening into postnatal care, strengthening partner- and community-based support, and expanding family-planning and counselling services are priority measures to reduce PPD burden in Addis Ababa and similar settings.

## Background

Major depressive disorder (MDD) is defined clinically by the presence of at least five depressive symptoms during a continuous two-week period, with one core symptom being depressed mood or markedly diminished interest or pleasure [1]. In the context of childbirth, the American Psychiatric Association specifies that postpartum depression (PPD) refers to an MDD episode beginning shortly after delivery (within about four weeks) [2]. Depression is a common global illness: World Health Organization (WHO) estimates indicate that roughly 280–300 million people worldwide suffer from it, and it is about 50% more prevalent in women than in men [3]. In particular, over 10% of women experience a depressive disorder during pregnancy or in the first year after birth [3,4]. Postpartum depression thus emerges in a vulnerable period and is the most frequent psychological disorder following childbirth [4,5]. A large meta-analysis found the worldwide prevalence of PPD to be about 17.2% [5]. However, this prevalence has not been static: during the COVID-19 pandemic, rates of perinatal depression rose sharply, with pooled estimates of PPD around 27–28% [6]. And prevalence estimates in some low-resource settings were substantially higher (often 20–40%), with some individual studies reporting prevalences up to ~80% during the pandemic [7]. In contrast, a recent multinational study reported that PPD prevalence declined to roughly 13.6% in the post-pandemic period [8]. In Ethiopia, systematic reviews likewise document a high burden: pooled analyses found about 22–23% of postnatal women screened positive for depression [9,10]. For example, a national meta-analysis estimated the prevalence of PPD at 22.9% [9], and a health-center study in Addis Ababa reported that 19.7% of mothers screened with the EPDS had depressive symptoms [2].

Several maternal and social factors predispose women to PPD. Younger maternal age, intimate partner violence, poverty, and lack of social support have all been linked to a higher risk of postpartum depression [4]. For instance, a community study in Sudan found that a history of domestic violence increased the odds of postnatal depression by more than sevenfold, whereas each additional year of maternal age reduced the risk slightly [11]. Low-income and under-resourced countries face additional challenges: WHO notes that over 75% of people with depression in low- and middle-income nations receive no treatment [12]. In such settings, socio-cultural stigma and weak health infrastructure often result in PPD being underdiagnosed and untreated. This “treatment gap” and lack of maternal mental health awareness mean that many affected mothers go without help, perpetuating the problem [13].

Postpartum depression has serious public health consequences. It undermines maternal well-being and the quality of infant care: depressed mothers often have difficulty bonding with and caring for their newborns, which can impair child growth and development [14]. PPD can also lead to adverse family outcomes, such as impaired parenting and increased family stress over time [9]. In recognition of this, experts have described PPD as a “serious public health problem” globally, with particularly harmful effects in developing countries [4]. Given its prevalence and impact, routine screening for depression during the postpartum period has been advocated. For example, Ethiopian public health researchers recommend integrating mental health screening into maternal care, urging that all postnatal clinics routinely assess mothers for depressive symptoms and provide appropriate referrals [2].

Despite growing recognition of postpartum depression as a major contributor to maternal morbidity, important evidence gaps remain in the Ethiopian context. Existing studies are largely concentrated in rural settings or tertiary hospitals, with limited data from urban primary-care facilities, where most postnatal care is routinely delivered. Moreover, much of the available Ethiopian and regional evidence was generated before or during the COVID-19 pandemic, a period marked by substantial disruptions to maternal health services and heightened psychosocial stressors [15]. As a result, there is limited understanding of the post-pandemic burden and correlates of postpartum depression in routine care settings following the end of the COVID-19 Public Health Emergency of International Concern. Addressing this gap is essential for informing the integration of mental health screening and support into standard postnatal services. Therefore, this study aimed to assess the prevalence of postpartum depression and its associated factors among postpartum mothers attending urban primary health centers in Addis Ababa, Ethiopia.

## Methods and materials

### Study design and period

We conducted an institution-based cross-sectional study from March 1 to April 30, 2024, to assess the prevalence and associated factors of postpartum depression (PPD) among mothers attending postnatal follow-up at health centers in Bole sub-city, Addis Ababa, Ethiopia. For the purposes of this study, the “post-COVID-19 era” is defined as the period after 5 May 2023, when the World Health Organization declared the end of the COVID-19 public health emergency of international concern [15].

### Study setting

Bole sub-city, located in the eastern part of Addis Ababa, has an estimated population of 378,104 (176,555 females). The area includes five health centers: Bole 17, Dil Fre, Wereda 13, Bole 17/20, and Bole Bulbula Wereda 12; each provides outpatient and maternity services, including antenatal care (ANC), labor and delivery, and postnatal follow-up. These centers serve diverse urban populations and maintain routine postnatal clinics.

### Study population

The source population comprised all postpartum mothers attending postnatal care (PNC) services at the selected health centers during the study period. The study population consisted of postpartum mothers attending these clinics who met the eligibility criteria and were selected through the sampling procedure.

### Inclusion criteria

Eligible participants were mothers aged ≥18 years who were between 2 weeks and 12 months postpartum at the time of interview and attending postnatal care services during the study period. Permanent residency in Bole sub-city was required to ensure consistency in follow-up context.

Participants were assessed at varying postpartum time points within this period, and postpartum duration at the time of interview was recorded for each participant and considered during analysis. Mothers were included from 2 weeks postpartum onward to reduce misclassification with transient postpartum blues, which typically resolve within the first 10–14 days after delivery [16].

### Exclusion criteria

Mothers who were not permanent residents of Bole sub-city and those who delivered outside the selected health centers were excluded.

### Sample size determination

The sample size was determined using a single population proportion formula, assuming a 95% confidence interval, 5% margin of error, and a prevalence (p) of PPD at 19.7% (from a previous Addis Ababa study). The calculated sample was 243, adjusted for various risk factors and non-response to a final sample size of 410, allocated proportionally across the five health centers. Participants were selected by systematic random sampling based on each center’s client volume (Table 1).

**Table 1:** Sample size calculation of the significant factors associated with postpartum depression among mothers receiving postnatal follow-up at health centers in Bole sub-city, Addis Ababa, Ethiopia March–April 2024 [2].

Variable Name	% outcome in the exposed group	AOR	n
**Occupational status**	18.4%	3.39	231
**Desired sex for the last baby**	17.6%	5.07	224
**Ever experienced the death of a baby**	75%	6.93	265
**Planned last pregnancy**	17.5%	3.08	288
**Negative life event during the last pregnancy**	69.2%	2.39	328
**Previous history of depression**	58.7%	5.08	373


$$\begin{array}{c} n\;\frac{{\left(z_{\alpha /\text{2}}\right)}^{\text{2}}\;\left(p(\text{1}-p)\right)}{d^{\text{2}}} \end{array}$$

Where;

- n: the number of participants to be interviewed,
- (Z α/2)2: standardized normal distribution value for the 95% CI, = 1.96,
- P: proportion of postpartum depression (19.7%) taken from a study conducted in Addis Ababa and
- d: margin of error taken as 5%.

$$\begin{array}{c} {\text{n}}_{\text{0}}=\;\frac{{\left(\text{1}.\text{9}\text{6}\right)}^{\text{2}}\;*\text{0}.\text{1}\text{9}\text{7}(\text{1}\;-\text{0}.\text{1}\text{9}\text{7}}{{\text{0}.\text{0}\text{5}}^{\text{2}}}-\text{2}\text{4}\text{3}.\text{0}\text{8}\sim \;\text{2}\text{4}\text{3} \end{array}$$

### Sampling procedure

We used systematic random sampling among postpartum mothers attending postnatal care services. For each health center, the required sample size was allocated proportionally based on expected postpartum clinic attendance during the data-collection period. The sampling interval (k) was calculated as k = N/n, where N was the estimated number of eligible postpartum mothers expected to attend the clinic during the study period and n was the allocated sample size for that facility. The value of N was obtained from postnatal clinic registers by calculating the average number of eligible postpartum visits per week and multiplying by the number of clinic weeks within the data-collection period.

All mothers presenting for postnatal care during data-collection hours were screened for eligibility prior to application of the sampling interval. To ensure a random start, the first participant was selected using a random number between 1 and k (lottery method) [17]. Thereafter, every k^th^ eligible mother was approached for participation as they attended the clinic. If a selected k^th^ mother did not meet eligibility criteria the next eligible mother was approached, and the systematic interval was maintained for subsequent selections. Participants were assessed at varying postpartum time points ranging from 2 weeks to 12 months after delivery. Postpartum duration at the time of interview was recorded for each participant and considered during analysis.

### Variables

The dependent variable was the presence of postpartum depression, defined as an EPDS score ≥11.

Independent variables included socio-demographic factors (age, marital status, education, occupation, income), obstetric factors (parity, pregnancy planning, mode of delivery, gestational age, delivery complications), psychosocial factors (social support, partner support, stressful life events), and clinical factors (history of depression, HIV status, substance use, infant birth weight, and gender preference).

### Comparative approach

In addition to estimating the prevalence of postpartum depression (PPD) in the post-COVID-19 era, we planned to compare our findings with published prevalence estimates from pre-pandemic and during-pandemic periods, both globally and in Ethiopia. Comparative figures were drawn from large meta-analyses [4–7]and key local studies conducted before and during COVID-19 [9,10]. This contextualization was intended to highlight potential changes in PPD burden following the official end of the COVID-19 public health emergency in May 2023.

### Operational definitions

- **Personal previous history of depression:** A past major depressive episode (MDD), defined by at least five Diagnostic and Statistical Manual of Mental Disorders, Fifth Edition (DSM-5) symptoms (including either depressed mood or loss of interest/pleasure) lasting at least two weeks [1].
- **Family and social support:**
  - ◦ *Social support*: Measured by Oslow social support scale-3 (OSSS-3) (score 3–8 = poor, 9–11 = moderate, 12–14 = good) [18].
  - ◦ *Family support*: Assessed with Family Adaptability and Cohesion Evaluation Scales (FACES IV) (12–15 = good, 7–11 = fair, 3–6 = poor) [19].
- **Substance abuse:** Any use of substances (e.g., khat, alcohol, cigarettes, or recreational drugs) causing impairment or distress [20].
- **Stressful life event during pregnancy:** Any significant event causing undue emotional or physical stress during pregnancy [2].
- **Parity:** Number of viable pregnancies; primiparous (1), multiparous (2–4), grand multiparous (≥5) [21].
- **Mode of delivery:** Spontaneous vaginal, induced vaginal, or cesarean [21].
- **Gestational age of delivery:** Preterm (<37 weeks), term (37–41 + 6 weeks), post-term (≥42 weeks) [21].
- **Pregnancy and delivery complications:** Presence of any complication (bleeding, infection, hypertension, perineal tear, prolonged/obstructed labor, stillbirth) [21].
- **Weight of the baby:**
  - ◦ Small for gestational age (SGA) (<10th percentile),
  - ◦ Appropriate for gestational age (AGA) (10th–90th percentile),
  - ◦ Large for gestational age (LGA) (>90th percentile) [22].
- **Partner support:** Measured by the Women Abuse Screening Tool (WAST-7); score ≥14 = good, < 14 = poor [23].
- **Postpartum depression:** Postpartum depression was assessed using the EPDS, a 10-item screening tool with each item scored from 0 to 3, yielding a total score range of 0–30. The EPDS was administered in Amharic through interviewer-administered face-to-face interviews by trained data collectors. Items were scored according to standard EPDS guidelines, including reverse scoring of appropriate items. A cutoff score of ≥11 was used to indicate probable postpartum depression, based on prior validation studies in similar settings. Item 10, which assesses thoughts of self-harm, was reviewed immediately at the time of administration. Participants endorsing this item or scoring above the cutoff were managed according to the predefined referral protocol described in the Ethics Statement [23].

### Data collection procedure

Data were collected using a structured, pre-tested, face-to-face interview questionnaire adapted from validated tools and relevant literature. The questionnaire included socio-demographic, obstetric, support, and depression-related items, with EPDS-10 as the primary measure of postpartum depression [16]. All study instruments were administered in Amharic. The questionnaires were translated from English into Amharic and independently back-translated into English by bilingual experts, with discrepancies reviewed and resolved.

The EPDS was administered by trained data collectors using a standardized interviewer-administered approach. Each item was read aloud to participants, and responses were recorded based on participants’ self-report. Scoring followed standard EPDS guidelines. Item 10, which assesses thoughts of self-harm, was reviewed immediately during the interview to allow prompt identification of participants requiring further support or referral, in accordance with the study’s ethical protocol.

All eligible mothers approached during the data-collection period consented to participate, and no eligible mother declined participation. Interviews were conducted in a private area of the clinic to support confidentiality and minimize social desirability bias.

Because the questionnaire included sensitive mental health and interpersonal items, data collectors received intensive training on standardized administration, neutral probing, confidentiality, and rapport-building. Supervisors and principal investigators performed daily checks to ensure completeness and consistency of the collected data.

### Data quality assurance

- Pre-testing was conducted on 10% of the sample at a comparable health center.
- Data collectors and supervisors received standardized training.
- A daily review of collected data for completeness and consistency was performed by supervisors and principal investigators.

### Data analysis

Data were entered into EpiData and analyzed using SPSS version 29. Descriptive statistics, including frequencies, percentages, means, and standard deviations, were used to summarize participant characteristics and the prevalence of postpartum depression. Bivariable logistic regression analyses were first performed to examine the association between each independent variable and postpartum depression. Variables with p < 0.25 in bivariable analysis, together with variables considered clinically or contextually relevant based on prior literature, were entered into the multivariable logistic regression model. Multivariable logistic regression was used to identify factors independently associated with postpartum depression while controlling for potential confounders. Adjusted odds ratios (AORs) with 95% confidence intervals (CIs) were reported. Multicollinearity was assessed using variance inflation factors, and model fit was evaluated using the Hosmer–Lemeshow goodness-of-fit test. Statistical significance was set at p < 0.05. Because some subgroup sizes were small, findings with wide confidence intervals were interpreted with caution.

### Ethics statement

Ethical approval was obtained from the Institutional Review Board of Myungsung Medical College Institutional Research Ethics Review Committee (MMC-IRERC), Addis Ababa, Ethiopia (Protocol No: PRO-170224, Approval No: MMC/EC/076/2024, dated 27 February 2024). Additional permissions were secured from the Addis Ababa Health Bureau, Bole Health Bureau, and participating health centers.

All participants provided written informed consent after being informed of the study’s aims, risks, and benefits. Confidentiality was maintained by assigning numeric identifiers and securely storing data. Interviews were conducted in private settings, and participants were informed that participation was voluntary and that declining or withdrawing would not affect their access to routine care.

Given the sensitive nature of postpartum mental health assessment, specific procedures were implemented to ensure participant safety. EPDS was administered by trained data collectors, and responses were reviewed in real time. Participants who scored ≥11 were provided with immediate supportive counseling by trained health professionals available at the health centers.

Participants who endorsed suicidal ideation (EPDS item 10) or exhibited marked psychological distress were identified immediately during the interview and referred on the same day to on-site mental health services or, where necessary, to the nearest psychiatric outpatient clinic for further evaluation and management. Referral was conducted through established clinical pathways within the participating facilities, ensuring continuity of care.

These procedures were implemented in accordance with the approved ethical protocol to ensure appropriate identification, support, and referral of participants with mental health needs.

## Results

A total of 410 mothers (2 weeks–12 months postpartum) participated (response rate 100%). The mean age was 28.8 years (SD 4.7). The prevalence of postpartum depression (EPDS ≥11) was 20.0% (82/410; 95% CI 16.4%–24.1%) (Table 2).

**Table 2:** Comparative prevalence of postpartum depression before, during, and after the COVID-19 pandemic.

Period	Source	Setting	Pooled prevalence (%)
Pre-pandemic	Woody et al., 2017 [4]	Global meta-analysis	17.2
During pandemic	Adrianto et al., 2022 [6]	Global meta-analysis	27–28
During pandemic	Usmani et al., 2021 [7]	Multi-country review	~26*
Post-pandemic	*This study*	Addis Ababa, Ethiopia	20.0 (95% CI 16.4%–24.1%)

*Reported range across studies: 7.0–80.8%.

### Socio-demographic related characteristics of respondents

Among the 410 participants, the mean age was 28.8 years (SD 4.7). Most were married (93.9%), 31.2% had completed secondary education, and 53.9% were unemployed. The mean monthly income was 7,141 ETB (SD 4,036). Sixteen participants (3.9%) reported being HIV positive, and 9 (2.2%) reported substance use during the indexed pregnancy (Table 3).

**Table 3:** Socio-demographic characteristics of mothers receiving postnatal follow-up at health centers in Bole sub-city, Addis Ababa, Ethiopia, during the period from March 01 to April 30, 2024.

Variable	Category	Frequency	Percent
**Age**	15-19	5	1.2%
	20-24	83	20.2%
	25-29	143	34.9%
	30-34	113	27.6%
	≥ 35	66	16.1%
**Marital status**	Married	385	93.9%
	Single	17	4.1%
	Divorced/Separated	8	2%
**Education**	Can’t read and write	60	14.6%
	Read and write	36	8.8%
	Elementary education	110	26.8%
	Secondary education	128	31.2%
	Tertiary education	76	18.5%
**Occupation**	Employed	189	46.1%
	Unemployed	221	53.9%
**Income**	< 5000	117	29.3%
	5000 - 9999	178	43.4%
	10000-14999	85	20.7%
	15000-19999	23	5.6%
	≥ 20,000	7	1.7%
**Substance use**	Yes	9	2.2%
	No	391	95.4%
	Don’t want to disclose	7	1.7%
**HIV status**	Positive	16	3.9%
	Negative	387	94.4%
	Don’t want to disclose	7	1.7%

### Obstetrics-related characteristics of respondents

Among the participants, 55.1% were multiparous and 63.7% reported that the pregnancy was planned. Stressors during pregnancy were reported by 32.9%, and 19.8% experienced delivery-related complications. Most participants delivered by spontaneous vaginal delivery (73.2%), 59.8% delivered preterm, and 73.2% of infants were appropriate for gestational age. Overall, 42.7% of mothers reported no preference for the sex of the baby, and 90.5% were breastfeeding at the time of the interview (Table 4).

**Table 4:** Obstetric characteristics of mothers receiving postnatal follow-up at health centers in Bole sub-city, Addis Ababa, Ethiopia, during the period from March 01 to April 30, 2024.

Variable	Category	Frequency	Percent
**Parity**	Primiparous	175	42.7%
	Multiparous	226	55.1%
	Grand multiparous	9	2.2%
**Planned Pregnancy**	Yes	261	63.7%
	No	149	36.3%
**Gestational Age**	Preterm	245	59.8%
	Term	159	38.8%
	Post-term	6	1.5%
**Mode of delivery**	Spontaneous vaginal delivery (SVD)	300	73.2%
	Induced labor	77	18.8%
	Cesarean Section (CS)	33	8%
**Delivery-related complications**	Yes	81	19.8%
	No	329	80.2%
**Wanted the gender of the baby**	Yes	155	37.9%
	No	80	19.5%
	Had no preference	175	42.7%
**Stressor during pregnancy**	Yes	135	32.9%
	No	275	67.1%
**Breast feeding**	Yes	371	90.5%
	No	39	9.5%
**Weight of the baby**	SGA	64	15.6%
	AGA	300	73.2%
	LGA	46	11.2%

### Depression and support related characteristics of respondents

**Fig 1:**
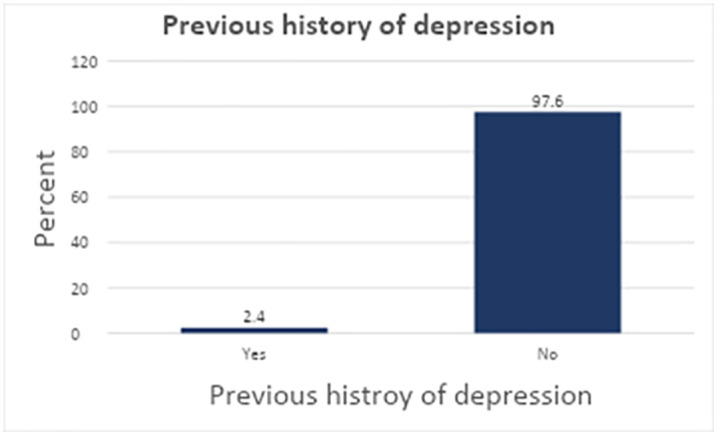
Mothers who had a previous history of depression now on postnatal follow-up at health centers in Bole sub-city, Addis Ababa, Ethiopia, during the period from March 01 to April 30, 2024.

Among the respondents, 14.4% reported having experienced symptoms of depression with onset before the indexed pregnancy. Among those who reported experiencing depressive symptoms, 2.4% had a previous history of depression (Fig 1). Among the mothers who were included in the current study, 93.9% (385 individuals) reported having good partner support. Similarly, 85.4% (350 individuals) stated that they had good social support. Furthermore, 87.6% (359 individuals) reported having a good relationship with their family.

### Prevalence of postpartum depression

**Fig 2:**
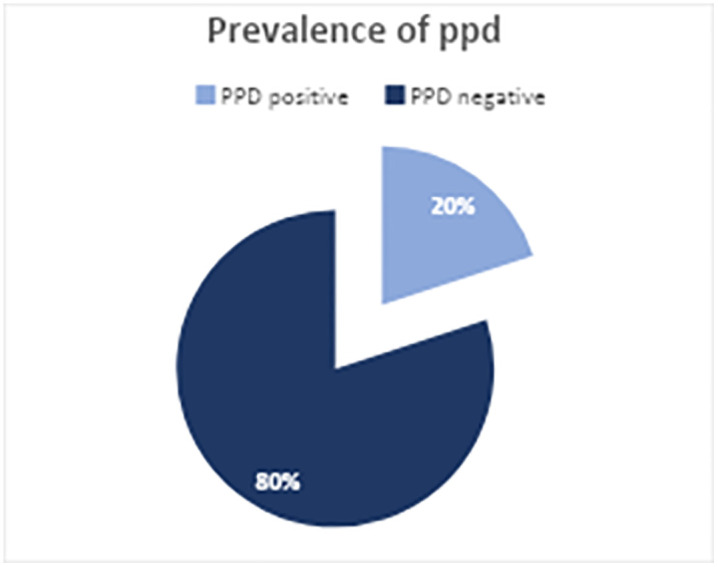
Prevalence of PPD of mothers receiving postnatal follow-up at health centers in Bole sub-city, Addis Ababa, Ethiopia, during the period from March 01 to April 30, 2024.

**Fig 3:**
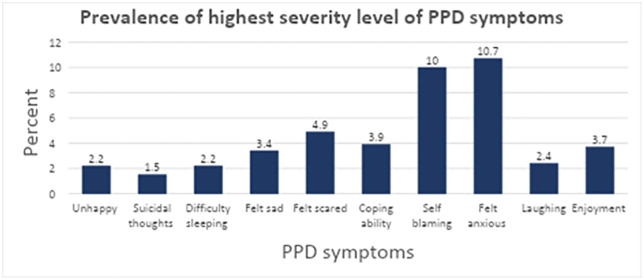
Prevalence of severe level of PPD symptoms of mothers receiving postnatal follow-up at health centers in Bole sub-city, Addis Ababa, Ethiopia, during the period from March 01 to April 30, 2024.

The prevalence of PPD was found to be 20% (Fig 2). Among the components used to assess PPD, anxiety was the most common symptom at the highest severity level (10.7%), whereas suicidal thoughts were the least common (1.5%) (Fig 3).

### Factors associated with postpartum depression

In multivariable logistic regression analysis, parity, planned pregnancy, partner support, social support, and mode of delivery remained significantly associated with postpartum depression. Primiparous mothers had higher odds of postpartum depression compared to grand multiparous mothers (AOR = 7.829; 95% CI 1.285–47.709), although the estimate was imprecise as reflected by the wide confidence interval. Mothers with unplanned pregnancies had higher odds of postpartum depression compared to those with planned pregnancies (AOR = 2.488; 95% CI 1.250–4.952). Poor partner support was associated with postpartum depression (AOR = 13.234; 95% CI 3.076–56.935), and low social support was also associated with increased odds (AOR = 3.311; 95% CI 1.126–9.737). Induction of labor was associated with higher odds of postpartum depression compared to spontaneous vaginal delivery (AOR = 2.729; 95% CI 1.128–6.603). Some estimates were associated with wide confidence intervals, indicating limited precision (Table 5).

**Table 5:** Bi-variable and multivariate binary logistic regression analysis for factors associated with postpartum depression attending postnatal follow-up at health centers in Bole sub-city, Addis Ababa, Ethiopia, during the period from March 01 to April 30, 2024. Some adjusted odds ratios were large and accompanied by wide confidence intervals, particularly for variables with small numbers of exposed participants, indicating limited precision of these estimates.

Variable	Category	PPD (+)	PPD (−)	COR (95% CI)	AOR (95% CI)	P-value
**Parity**	Primiparous	26	175	7.163 (1.084–28.451)	7.829 (1.285–47.709)	0.026*
	Multiparous	51	149	1.670 (0.993–2.810)	1.771 (0.840–3.736)	0.133
	Grand multiparous	5	4	1 (ref)	1 (ref)	—
**Marital status**	Married	9	8	1 (ref)	1 (ref)	—
	Single	69	316	5.152 (1.920–13.829)	3.092 (0.542–17.624)	0.204
	Divorced/Separated	4	4	4.580 (1.118–18.761)	0.861 (0.107–6.952)	0.888
**Education**	Cannot read and write	20	40	1 (ref)	1 (ref)	—
	Read and write	7	29	0.483 (0.180–1.292)	0.505 (0.136–1.873)	0.307
	Primary education	28	82	0.683 (0.344–1.358)	0.744 (0.304–1.822)	0.517
	Secondary education	20	108	0.370 (0.181–0.760)	0.501 (0.193–1.302)	0.156
	Tertiary education	7	69	0.203 (0.079–0.522)	0.404 (0.094–1.734)	0.223
**Income (ETB)**	<5,000	35	82	1 (ref)	1 (ref)	—
	5,000–9,999	33	145	0.533 (0.308–0.922)	0.880 (0.423–1.829)	0.423
	10,000–14,999	7	78	0.210 (0.088–0.501)	0.431 (0.145–1.283)	0.131
	15,000–19,999	5	18	0.651 (0.224–1.891)	1.429 (0.344–5.931)	0.623
	≥20,000	2	5	0.937 (0.173–5.063)	3.444 (0.368–32.218)	0.278
**Planned pregnancy**	Yes	31	230	1 (ref)	1 (ref)	—
	No	51	98	0.259 (0.156–0.429)	2.488 (1.250–4.952)	0.009*
**Stressor during pregnancy**	Yes	40	95	1 (ref)	1 (ref)	—
	No	42	233	2.336 (1.425–3.830)	0.617 (0.311–1.223)	0.167
**Weight of baby**	SGA	17	47	1 (ref)	1 (ref)	—
	AGA	63	237	0.735 (0.395–1.367)	0.927 (0.399–2.157)	0.861
	LGA	2	44	0.126 (0.027–0.576)	0.265 (0.046–1.537)	0.139
**Substance use**	Yes	7	2	1 (ref)	1 (ref)	—
	No	71	320	5.250 (0.698–39.476)	0.128 (0.011–1.496)	0.101
	Do not disclose	4	6	0.333 (0.092–1.210)	0.048 (0.001–2.016)	0.111
**Partner support**	Good	62	323	1 (ref)	1 (ref)	—
	Poor	20	5	20.839 (7.537–57.615)	13.234 (3.076–56.935)	0.001*
**Family relationship**	Good	62	297	1 (ref)	1 (ref)	—
	Fair	12	23	2.499 (1.181–5.289)	0.521 (0.143–1.900)	0.323
	Poor	8	8	4.790 (1.732–13.251)	3.705 (0.824–16.656)	0.088
**Social support**	Good	53	297	1 (ref)	1 (ref)	—
	Fair	20	23	4.873 (2.502–9.491)	3.311 (1.126–9.737)	0.030*
	Poor	9	8	6.304 (2.328–17.072)	3.103 (0.620–15.521)	0.168
**Occupation**	Employed	21	146	1 (ref)	1 (ref)	—
	Unemployed	61	182	1.520 (0.536–4.314)	1.974 (0.830–4.692)	0.124
**Gestational age**	Preterm	54	191	1 (ref)	1 (ref)	—
	Term	26	133	0.691 (0.412–1.160)	0.791 (0.404–1.550)	0.495
	Post-term	2	4	1.769 (0.315–9.916)	5.826 (0.654–51.872)	0.114
**Mode of delivery**	SVD	58	242	1 (ref)	1 (ref)	—
	Induction	20	57	1.464 (0.816–2.626)	2.729 (1.128–6.603)	0.026*
	Cesarean section	4	29	0.576 (0.195–1.701)	0.328 (0.064–1.677)	0.181
**Delivery complications**	Yes	20	61	1 (ref)	1 (ref)	—
	No	62	267	0.708 (0.398–1.259)	1.319 (0.491–3.542)	0.583
**HIV status**	Positive	5	11	1 (ref)	1 (ref)	—
	Negative	72	315	0.503 (0.169–1.492)	0.791 (0.139–4.506)	0.792
	Do not disclose	5	2	5.500 (0.782–38.698)	12.237 (0.797–187.819)	0.072
**Preferred sex of baby**	Yes	25	130	1 (ref)	1 (ref)	—
	No	18	62	1.510 (0.767–2.972)	0.764 (0.289–2.021)	0.588
	Do not disclose	39	136	1.491 (0.855–2.602)	1.300 (0.614–2.754)	0.493

## Discussion

In this post-COVID-19 period, one in five mothers (20.0%) in our sample screened positive for PPD, an estimate that lies between pre-pandemic and pandemic peaks. Pre-pandemic meta-analyses estimated global PPD prevalence around 17% [8], whereas pooled studies during COVID-19 found much higher rates (27–28%) [6]. Our 20% (95% CI 16.4–24.1) is slightly above those earlier baselines but below the pandemic peaks, which may reflect a lower prevalence compared with pandemic-era estimates, although direct temporal comparisons cannot be made due to the cross-sectional design. Notably, this rate closely matches recent local findings, for example, a 2021 Addis Ababa study reported 19.7% [2], and is similar to Ethiopia’s pooled prevalence (~22.9%) [9]. Thus, even after the pandemic emergency ended, PPD remains a substantial public health issue in Addis Ababa. Some adjusted estimates were accompanied by wide confidence intervals, indicating limited precision, particularly for variables with small subgroup sizes. These findings should therefore be interpreted as indicators of association rather than precise estimates of effect size, and causal inferences cannot be made due to the cross-sectional design.

The observed associations between postpartum depression and psychosocial and obstetric factors, including unplanned pregnancy and limited social support, are consistent with findings from prior Ethiopian and regional studies [9,24]. Previous studies have similarly reported higher odds of postpartum depression among women with a history of depressive symptoms, unplanned pregnancies, limited social support, and exposure to interpersonal stressors, supporting the direction of associations observed here [14]. However, the literature also shows variability in the magnitude of these associations across settings, and some studies report weaker or non-significant relationships for certain obstetric characteristics [25]. Such inconsistencies may reflect differences in study design, postpartum time windows, measurement tools, and contextual factors, underscoring the need for cautious interpretation and context-specific application of findings.

Social support was also critical. Women reporting poor social support during pregnancy had roughly three times the odds of PPD. This finding aligns with previous studies showing that inadequate support is a potent predictor of postpartum depression [2,26]. For example, Roumieh et al. describe lack of support in pregnancy as a “relatively potent” risk factor for PPD [26]. Wake et al. similarly cite poor social support as a significant contributor to postpartum symptoms [2]. In the absence of adequate emotional and practical help, new mothers may feel isolated and overwhelmed, which can exacerbate stress and precipitate depressive symptoms after childbirth [14].

Likewise, partner support had a major influence: women with minimal spousal or partner support were far more likely to be depressed. This pattern echoes findings from other settings that highlight marital or partner relationship quality as a key determinant of maternal mood [27,28]. Ongeri et al., for example, found that intimate partner conflict increased PPD risk by more than sevenfold [27]. Ruan et al. emphasize that a supportive partner provides crucial emotional stability and practical assistance that help buffer against the stresses of new motherhood [28]; conversely, lack of partner support is associated with increased isolation and distress [27,28]. In our study, poor partner support was associated with over 13-fold higher odds of PPD, underlining how much a caring partner can protect maternal mental health.

Pregnancy planning was another important factor. Mothers with an unplanned pregnancy had higher odds of PPD compared to those with planned conceptions. This is in line with prior reports: for instance, Wake et al. found that unintended pregnancy tripled the odds of PPD (AOR ≈ 3.1) [2], and Kerie et al. observed an even higher risk (AOR ≈ 4.5) for unplanned pregnancies in southwest Ethiopia [29]. Unplanned or unwanted pregnancies may leave women feeling unprepared, financially and emotionally, for motherhood, intensifying stress and anxiety that can contribute to depressive symptoms after birth [30].

Finally, labor induction was associated with higher PPD risk in our cohort. Although direct evidence on induction is limited, this finding is consistent with broader literature on birth interventions. A recent meta-analysis found that women delivering by cesarean section (especially emergency C-section) faced about a 1.5-fold higher odds of postpartum depression compared to those having spontaneous vaginal births [31]. Induction of labor can similarly change a woman’s birth experience. For example, it may increase the likelihood of urgent C-section or longer, more painful labor, which has been associated in previous studies with more complex delivery experiences and increased emotional distress [32]. In our data, induction was associated with roughly three times the odds of PPD, perhaps reflecting these indirect effects (e.g., complicated delivery, unmet birth expectations) that have been linked to postpartum depressive outcomes [31].

In summary, the 20% PPD prevalence we observed is below the very high rates seen during COVID-19 but remains elevated relative to pre-pandemic norms [4]. The risk factors we identified – first-time motherhood, low social/partner support, unintended pregnancy, and more-interventive birth – largely mirror those found before and during the pandemic [26,31,33]. These findings suggest that key psychosocial risk factors for postpartum depression remain present in the post-pandemic context, but maternal vulnerabilities and psychosocial stressors persist. Strengthening family and community support for new mothers, improving access to family planning, and managing labor interventions remain important priorities [34]. Our findings underscore the need for routine mental health care within postnatal services. In fact, as Ethiopian health experts have noted, routine PPD screening and linkage to care are often neglected [2], so integrating mental health into maternal health services is crucial.

### Recommendations

- Routine screening for postpartum depression should be integrated into postnatal care services, with appropriate referral pathways for identified cases.
- Enhance partner and social support. Encourage family- and community-based support programs and involve partners through counseling and education to help buffer maternal stress.
- Expand family planning and counseling. Reducing unintended pregnancies through effective family planning can lower stress on new mothers.
- Target first-time mothers. Provide extra education and psychosocial support to primiparous women, who may need additional guidance and reassurance during their first postpartum experience.
- Extend maternal mental health screening and counseling to antenatal care (ANC) services, particularly for first-time mothers and women with unplanned pregnancies, as many key drivers of postpartum depression originate during pregnancy and can be effectively addressed through early identification, psychosocial support, and family-planning–linked interventions through maternal and child health programs within public health centers, supported by regional health authorities.

## Conclusion

In this post-COVID-19 snapshot (data collected March–April 2024, after WHO’s May 5, 2023, declaration), one in five mothers attending postnatal clinics in Bole sub-city screened positive for PPD (20.0%, 95% CI 16.4–24.1). This prevalence sits between pre-pandemic pooled estimates (~17.2%) and pandemic peaks (≈27–28%), which is lower than pandemic-era estimates but remains higher than some pre-pandemic reports. Importantly, post-pandemic correlates mirror persistent vulnerabilities: primiparity (AOR ≈ 7.8), unplanned pregnancy (AOR ≈ 2.5), low social support (AOR ≈ 3.3), poor partner support (AOR ≈ 13.2), and labor induction (AOR ≈ 2.7). Although some estimates are imprecise, the pattern indicates that persistent psychosocial risk factors remain evident in the post-pandemic context. For the post-COVID era, health systems should prioritize routine PPD screening in postnatal care with clear referral pathways, scale partner- and community-based support interventions, strengthen family-planning services to reduce unintended pregnancies, and offer targeted education and psychosocial support to first-time mothers. Given the cross-sectional design and use of EPDS as a screening tool, further longitudinal and diagnostic studies are needed to monitor trends and guide tailored post-pandemic maternal mental health strategies.

### Limitations

This study has several limitations that should be considered when interpreting the findings. First, the cross-sectional design provides only a snapshot of postpartum depression prevalence and associated factors in the post-COVID-19 period, limiting our ability to establish causal relationships or confirm changes in prevalence over time. Second, the absence of a direct pre-pandemic and post-pandemic comparative design restricts inferences about temporal trends and pandemic-related impacts. Our reliance on facility-based sampling from selected health centers may introduce selection bias, as women attending postnatal clinics might differ systematically from those who do not seek care, potentially affecting the generalizability of results to the broader population. Third, no COVID-19-specific variables, such as infection history, pandemic-related stressors, or changes in healthcare access, were collected, which limits our capacity to directly attribute observed postpartum depression rates or risk factors to pandemic effects. Finally, although validated instruments were used to assess postpartum depression, social support, family functioning, and partner support, internal consistency measures (e.g., Cronbach’s alpha) were not recalculated for the current sample. However, all tools employed have demonstrated acceptable reliability and validity in previous studies conducted in Ethiopia or similar low-resource settings. The multivariable model included some exposure categories with small cell counts, which may have resulted in wide confidence intervals and reduced precision for certain adjusted estimates. Future longitudinal and community-based studies incorporating pandemic-specific measures are warranted to better understand the evolving impact of COVID-19 on maternal mental health. Future research should consider extending the assessment period for postpartum depression beyond one year after childbirth, potentially up to 18–24 months, to capture late-onset and persistent depressive symptoms that may not be detected during routine postnatal care. Longitudinal designs following women from pregnancy through extended postpartum periods would provide more accurate estimates of PPD trajectories and inform the optimal timing of screening and intervention strategies in low-resource settings.

## Supporting information

S1 Data De-identified dataset used for the analysis. (SAV)

## Abbreviations

AGA: Appropriate for gestational age
ANC: Antenatal care
AOR: Adjusted odds ratio
CS: Cesarean section
DSM-5: Diagnostic and Statistical Manual of Mental Disorders, Fifth Edition
ETB: Ethiopian Birr
EPDS: Edinburgh Postnatal Depression Scale
FACES IV: Family Adaptability and Cohesion Evaluation Scales (FACES IV)
HIV: Human immunodeficiency virus
MDD: Major depressive disorder
OSSS-3: Oslo Social Support Scale-3
PPD: Postpartum depression
SGA: Small for gestational age
SVD: Spontaneous vaginal delivery
SD: Standard deviation
SPSS: Statistical Package for the Social Sciences
WAST-7: Women Abuse Screening Tool-7
WHO: World Health Organization
